# Factors associated with sarcopenia in older people with Alzheimer’s disease: a cohort study in a public outpatient clinic from the South of Brazil

**DOI:** 10.1002/alz.094618

**Published:** 2025-01-09

**Authors:** Lindsey Mitie Nakakogue, Marcos Aparecido Sarria Cabrera, Clara Spinosa Zanchetta, Renan Hayami Obata

**Affiliations:** ^1^ Pontifical Catholic University, Londrina, Parana Brazil; ^2^ State University of Londrina, Londrina, Parana Brazil; ^3^ Pontifical Catholic University, Londrina, Paraná Brazil

## Abstract

**Background:**

The association of Alzheimer’s disease (AD) and sarcopenia diagnoses is quite common, but frequently sarcopenia is underdiagnosed in this population and it may be contributing to the worsening of functionality in the person with AD.

**Method:**

Fifty‐five adults over age 60 from a public outpatient clinic in Londrina – Parana‐ Brazil diagnosed with Alzheimer’s disease were followed during the years 2022 and 2023. They were subjected to a sociodemographic questionnaire, BMI assessment, cognitive assessment using the MMSE, functionality assessment using the Katz Index and to a sarcopenia screening test SARC‐F +CC. They were evaluated at the beginning, after 4 and 8 months.

**Result:**

Of the fifty‐five older patients evaluated, 60% were female, 47.3% were between 70 and 79 years old, 43.6% had less than 4 years of schooling, 98.2% had mild Alzheimer’s (CDR I) and 25.5% had a BMI < 22 kg/m ^2^ in the beginning of the assessment. Only 18.2% were smokers, but few practiced physical activity regularly (21.8%). The majority had a diagnosis of Arterial Hypertension (69.1%). Almost half used five or more medications regularly (43.6%) and 36.4% used some medication with anorectic potential. Regarding the treatment of Alzheimer’s disease, 54.5% were using donepezil. Of the 55 older patients analyzed, 15 (27.3%) had probable sarcopenia at the end of 8 months of follow‐up. There was no association between the diagnosis of sarcopenia and gender, but the older the age, the greater the risk of sarcopenia, a risk of 1.091 (1.018‐ 1.170, p = 0.013). There was an association between the reduction in calf circumference and sarcopenia, with a CC < 33‐34cm risk of 9.391 (2.973‐29.664, p<0.001). In relation to patients without sarcopenia, those with probable sarcopenia had a reduction in BMI (p = 0.051). Older patients with probable sarcopenia had more falls, a risk of 1.744 (1.239‐2.456) and worsened functionality, a risk of 1.465 (1.078‐1.991) compared to older patients without sarcopenia (95% CI, p<0.001).

**Conclusion:**

The incidence of sarcopenia in older people with Alzheimer’s disease is high and should be investigated during the follow‐up of every older person to prevent negative outcomes such as falls and loss of functionality.

